# Symbiotic AI and equitable digital health: toward trustworthy and inclusive health ecosystems

**DOI:** 10.3389/fdgth.2026.1885289

**Published:** 2026-07-30

**Authors:** Rune Johan Krumsvik, Vegard Slettvoll

**Affiliations:** 1 Faculty of Psychology, University of Bergen, Bergen, Norway; 2 Faculty of Medicine, University of Bergen, Bergen, Norway

**Keywords:** digital health equity, health empowerment, implementation science, resilient health ecosystems, rural healthcare, symbiotic AI, telemedicine, trustworthy AI

## Abstract

Digital health has accelerated rapidly through advances in telemedicine, wearable technologies, artificial intelligence (AI), remote monitoring, and interoperable healthcare infrastructures. Despite these developments, substantial inequities persist among underserved populations, including rural and remote communities, older adults, low-socioeconomic groups, and geographically isolated populations exposed to infrastructure disruptions caused by avalanches, landslides, storms, flooding, or prolonged power outages. Current digital health approaches frequently emphasize technological innovation and connectivity while underestimating the importance of trust, contextual adaptation, resilience, health literacy, and human guidance. This *Perspective* argues that equitable digital health requires a shift from isolated AI tools in primary health services toward resilient *symbiotic health ecosystems* in which clinicians, patients, caregivers, communities, and AI systems collaboratively support healthcare delivery. Building on emerging research on symbiotic intelligence, health empowerment, telemedicine, rural resilience, and trustworthy AI, we propose a conceptual perspective in which calibrated human–AI collaboration becomes central to equitable healthcare delivery. The article discusses how resilient and locally adaptive infrastructures—including telemedicine, wearable monitoring, low-risk diagnostic technologies, local AI systems, backup energy systems, drones for medicine delivery, local Wi-Fi preparedness, and community-supported transport models—may strengthen healthcare preparedness and continuity in underserved and disrupted contexts. Although several examples are drawn from Norway and rural Nordic contexts, the conceptual framework is intended to be transferable to underserved populations globally. Finally, this Perspective highlights the need for implementation-oriented, equity-centered, and epistemically transparent approaches to future digital health ecosystems, exemplified through five models.

## Introduction

Digital health has expanded substantially during the last decade through advances in telemedicine, wearable technologies, artificial intelligence (AI), remote monitoring systems, and interoperable healthcare infrastructures. These developments have introduced new opportunities for healthcare accessibility, preventive medicine, continuity of care, and patient empowerment. The rapid emergence of generative AI and large language models has further intensified discussions concerning AI-supported triage, decision-support systems, clinical documentation, personalized health guidance, and patient communication.

At the same time, substantial inequities continue to shape healthcare access and outcomes among underserved populations. Rural and remote communities, older adults, low-socioeconomic groups, individuals with limited digital literacy, and geographically isolated populations remain disproportionately vulnerable to healthcare disparities. Importantly, these inequities are not solely related to technological access. Rather, they also involve issues related to trust, health literacy, accessibility, cultural adaptation, infrastructure resilience, and continuity of professional healthcare support.

Petretto et al. (1)'s scoping review argued that digital health inequities increasingly extend into digital healthcare environments, where unequal access to technologies, literacy, and infrastructure may reinforce existing healthcare disparities. Similarly, the World Health Organization (2)'s scoping review emphasized that equity should become a central dimension in the regulation, implementation, and evaluation of digital health systems. Emerging evidence also suggests that AI-supported healthcare systems may improve healthcare accessibility while simultaneously creating new challenges related to algorithmic bias, trustworthiness, and unequal implementation across populations (3).

A particularly underexplored dimension concerns healthcare resilience under conditions of infrastructural disruption. Many contemporary digital health systems implicitly assume stable broadband connectivity, cloud infrastructures, electricity supply, transportation systems, and institutional healthcare access. However, such assumptions may not hold in rural or disaster-prone regions where avalanches, landslides, storms, flooding, or prolonged power outages may significantly disrupt healthcare delivery and communication systems. In such contexts, equitable digital health becomes inseparable from health empowerment, preparedness, local resilience, and adaptive healthcare infrastructures.

Current debates surrounding AI in healthcare are frequently characterized by polarized narratives. On the one hand, AI is portrayed as transformative and potentially revolutionary for healthcare systems. On the other hand, concerns related to hallucinations, algorithmic bias, privacy, misinformation, overreliance, and dehumanization contribute to scepticism toward AI implementation. This polarized discourse may overlook a more productive perspective in which AI is understood neither as a replacement for healthcare professionals nor merely as a technological risk, but rather as part of a collaborative and context-sensitive healthcare ecosystem.

This article is presented as a Perspective rather than a narrative review and argues that equitable digital health requires a shift toward *symbiotic AI ecosystems*, where healthcare professionals, patients, caregivers, communities, and AI systems collaboratively support trustworthy and context-aware healthcare delivery. Building on emerging research related to symbiotic intelligence, health empowerment, telemedicine, rural resilience, and AI-supported healthcare systems, this article proposes that future digital health ecosystems should prioritize not only technological innovation, but also trust, resilience, health empowerment, local adaptability, implementation feasibility, and epistemic transparency.

For the purpose of this Perspective, five concepts are central: symbiotic AI, symbiotic intelligence, epistemic transparency, health empowerment, and resilient health ecosystems. The concept of *symbiotic intelligence*, which constitutes a central theoretical foundation of this Perspective, is defined and elaborated upon in a subsequent section of the article.

*Symbiotic AI* refers to a form of human–AI collaboration characterized by continuous co-interpretation, co-learning, co-decision-making, and mutual adaptation among humans, AI systems, organizations, and communities. Unlike task-oriented approaches such as human-in-the-loop systems, symbiotic AI emphasizes ecosystem-level interactions in which human and technological capabilities evolve together over time (4–6).

*Epistemic transparency* refers to the extent to which the assumptions, limitations, uncertainty, data sources, and reasoning processes underlying AI-generated outputs are understandable and interpretable to users. In healthcare, epistemic transparency is essential for calibrated trust, informed decision-making, and responsible human oversight of AI-supported systems (7, 8).

*Health empowerment* refers to individuals' capacity to access, understand, evaluate, and use health information and resources to make informed decisions about their own health and well-being. Within digital health ecosystems, empowerment involves not only information access but also the ability to critically interpret and act upon information provided through healthcare professionals, digital technologies, and AI-supported systems (2, 9).

*Resilient health ecosystems* are healthcare systems capable of maintaining essential functions, adapting to disruptions, and sustaining continuity of care under changing or adverse conditions. Such ecosystems integrate healthcare services, communities, infrastructures, technologies, governance structures, and preparedness mechanisms to support equitable healthcare delivery during both routine operations and periods of crisis or infrastructure disruption (2).

## Digital health inequities beyond technological access

Digital health inequities are frequently discussed primarily in relation to technological infrastructure, including broadband access, device availability, and Internet connectivity. Although these dimensions remain important, international research increasingly demonstrates that digital health equity is a multidimensional phenomenon shaped by broader social, educational, cultural, economic, and institutional factors (1).

The concept of *digital health equity* has emerged to describe how healthcare inequities increasingly extend into digital environments, including telemedicine, AI-supported healthcare systems, e-health platforms, and remote monitoring infrastructures. While digital technologies may reduce geographic barriers and improve healthcare accessibility, they may simultaneously create new forms of exclusion among populations with limited digital literacy, inadequate infrastructure, economic constraints, or low institutional trust.

Older adults may experience usability barriers and difficulties navigating digital interfaces, while rural populations may face unstable connectivity, long travel distances, and limited access to specialized healthcare personnel. Similarly, linguistic minorities and culturally diverse populations may encounter communication barriers when healthcare technologies are designed primarily for dominant language groups and standardized healthcare pathways. Men, especially those residing in rural and non-urban areas, often demonstrate lower levels of healthcare utilization, which may contribute to delayed help-seeking and reduced access to preventive healthcare services (10).

Importantly, digital health inequities are often reinforced by hidden assumptions embedded within healthcare technologies and implementation strategies. Many digital health systems are designed around urban and institution-centered models that presuppose stable infrastructure, uninterrupted cloud connectivity, and immediate access to healthcare institutions. Such assumptions may unintentionally marginalize populations living in geographically isolated or disaster-prone regions.

Osonuga et al. (3) highlighted that AI-enhanced telemedicine may improve healthcare accessibility in underserved regions through earlier intervention, reduced travel burden, and improved continuity of care. More specifically they found that telemedicine platforms reduced time to appropriate care by 40% in rural areas, only 15% of healthcare AI tools include community engagement in development, 85% of AI health equity studies tracked outcomes for less than 12 months, algorithmic bias leads to 17% lower diagnostic accuracy for minority patients, digital divide excludes 29% of rural adults from AI-enhanced healthcare tools. Therefore, the authors emphasized risks related to algorithmic bias, underrepresentation in datasets, and exclusion of populations with limited digital competence. These concerns are particularly important in healthcare systems increasingly relying on digital infrastructures and AI-supported communication systems.

Consequently, equitable digital health cannot solely be understood as technological access. Rather, it involves the capacity of healthcare ecosystems to remain trustworthy, adaptive, inclusive, and functional across diverse social and infrastructural conditions. This also implies that implementation science, health literacy, and contextual adaptation become central dimensions of future digital health strategies.

Within this context, *health empowerment* represents an especially important concept. Krumsvik et al. (9) suggested that AI-supported healthcare communication and decision-support systems may strengthen patient engagement, improve access to understandable health information, and lower barriers for seeking healthcare support. Yet empowerment depends not only on information access, but also on individuals' ability to interpret, contextualize, and critically evaluate AI-generated outputs. This highlights the importance of calibrated trust, human oversight, and epistemic transparency in future digital health ecosystems.

## Generative AI, trust, and symbiotic intelligence

The rapid development of generative AI has intensified discussions concerning the future role of AI in healthcare systems. Generative AI systems may support healthcare professionals through clinical documentation, triage assistance, translation, health communication, predictive analytics, and decision-support mechanisms. Emerging evidence suggests that AI-enhanced healthcare systems may contribute to improved healthcare accessibility and reduced geographic inequities in primary care settings (3).

At the same time, concerns related to reliability, hallucinations, interpretability, algorithmic bias, and overreliance remain substantial. Particularly in healthcare contexts, misleading or inaccurate outputs may have serious consequences for vulnerable populations with limited access to professional healthcare guidance. These concerns have intensified ongoing debates regarding trustworthiness, accountability, and ethical implementation of AI in healthcare.

**Figure 1:**
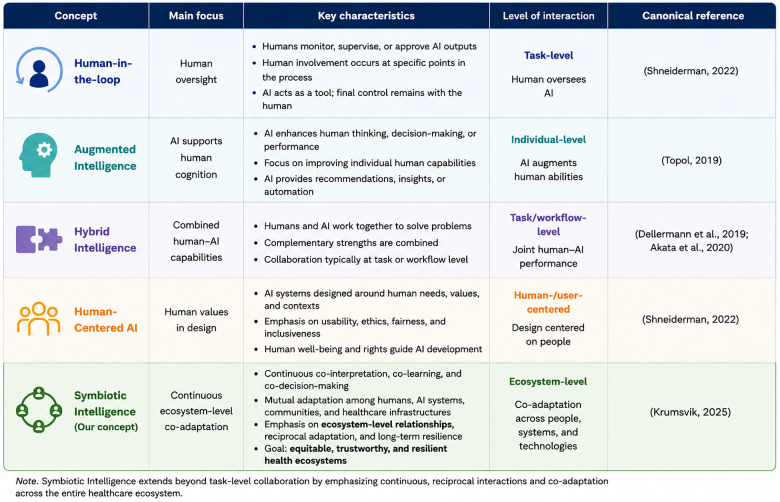
Symbiotic intelligence: comparison with related concepts.

Rather than framing AI as either inherently transformative or inherently dangerous, the concept of *symbiotic intelligence* may provide a more productive perspective*. Symbiotic Intelligence* builds upon earlier work on Human-Centered AI (11), Hybrid Intelligence (5, 6), and Augmented Intelligence (12), but extends these approaches by emphasizing ecosystem-level co-adaptation among humans, communities, healthcare systems, and AI technologies. We define symbiotic intelligence as a form of human–AI collaboration characterized by continuous co-interpretation, co-learning, co-decision-making, and mutual adaptation among humans, AI systems, communities, and healthcare infrastructures. Unlike related concepts such as augmented intelligence, hybrid intelligence, human-centered AI, and human-in-the-loop systems, symbiotic intelligence emphasizes ecosystem-level relationships in which human, technological, organizational, and societal actors continuously interact and adapt over time. Figure 1 illustrates a comparison of symbiotic intelligence with related concepts.

From this perspective, the primary value of AI does not emerge from automation alone, but from carefully calibrated human—AI collaboration. AI systems may augment healthcare professionals’ situational awareness, analytical capacity, and communication support while still relying on clinicians’ contextual interpretation and ethical responsibility.

A broader implication of this discussion concerns the growing role of generative AI as a healthcare navigation and support layer within increasingly strained healthcare systems. According to a recent non-peer-reviewed report published by OpenAI (13), preliminary findings from the United States suggests that generic generative AI is increasingly being used (based on private initiatives outside the health system) as a low-threshold healthcare support mechanism, particularly in underserved and geographically isolated regions (13). Notably, rural areas and so-called “hospital deserts” appear to use generative AI for health-related purposes more frequently relative to urban populations. This development may partly reflect structural challenges within healthcare systems, including limited healthcare accessibility, workforce shortages, and economic barriers. In the United States, approximately 8% of the population remains without health insurance, while a majority of Americans perceive the healthcare system as facing significant systemic challenges (13).

The report further highlighted that healthcare has become one of the largest use domains for generative AI globally. More than 5% of all ChatGPT interactions worldwide reportedly concern health-related questions, corresponding to more than 40 million health-related interactions daily (13). Moreover, approximately one in four ChatGPT users ask health-related questions on a weekly basis, suggesting that generative AI increasingly functions as a low-threshold and widely accessible health information service.

Importantly, the report emphasized that AI is frequently used when traditional healthcare services are unavailable rather than as a replacement for healthcare professionals. Approximately seven out of ten health-related AI conversations reportedly occur outside ordinary clinical opening hours, suggesting that generative AI increasingly serves as a complementary support layer during periods when formal healthcare access is limited (13). Similarly, more than 580,000 weekly health-related AI interactions reportedly originate from areas located more than 30 minutes from the nearest hospital, further illustrating how AI-supported systems may partly compensate for geographic healthcare inequities.

The findings also suggest that generative AI increasingly functions as a navigation and interpretation tool within complex healthcare systems. According to OpenAI (13), between 1.6 and 1.9 million weekly AI interactions concern health insurance issues alone, including questions related to pricing, coverage, denials, appeals, and patient rights. This highlights how AI systems may support not only medical information access, but also broader forms of health empowerment and healthcare system navigation. However, such findings should be interpreted cautiously because independent peer review has not yet been conducted.

These developments align closely with the concept of *symbiotic AI ecosystems* proposed in this article, where AI functions not as a replacement for healthcare professionals, but as a complementary, *context-sensitive*, and human-supervised support mechanism embedded within broader healthcare infrastructures. At the same time, the increasing societal dependence on generative AI for health-related support also underscores the importance of epistemic transparency, trustworthy implementation, equitable access, and clear governance frameworks capable of reducing risks related to misinformation, overreliance, and unequal digital health literacy (13).

Recent exploratory studies illustrate this perspective in practice. Krumsvik et al. (14) demonstrated how AI-supported systems in dental trauma diagnostics may strengthen clinical assessment without replacing professional expertise, thereby contributing to forms of hybrid expertise combining computational support with human judgment. Similarly, Krumsvik et al. (9) argued that AI-supported health empowerment initiatives may lower thresholds for seeking healthcare guidance and improve access to understandable health information, while still requiring human oversight and contextual adaptation.

This perspective aligns with broader international discussions concerning Hybrid Intelligence, Human-Centered AI, etc. and responsible clinician—AI collaboration and interpretable AI systems in healthcare (5–7, 12, 15). The World Health Organization (2) emphasized that trustworthy AI implementation requires transparency, accountability, and context-sensitive governance frameworks capable of protecting vulnerable populations from unintended harms.

Importantly, trustworthy AI systems require not only technical accuracy, but also *epistemic transparency* (8) Patients and healthcare professionals must understand the limitations, uncertainty, and contextual dependency of AI-generated outputs. Trustworthy healthcare ecosystems therefore depend on calibrated trust rather than blind reliance or categorical rejection of AI systems.

Symbiotic AI ecosystems may also contribute to more inclusive healthcare delivery through multilingual communication support, adaptive health information, and low-threshold healthcare guidance. However, such systems must be developed through equity-centered implementation approaches involving community participation, cultural adaptation, and accessibility-oriented design. Without these considerations, AI systems risk reproducing existing inequities through biased datasets, inaccessible interfaces, or technologically unrealistic implementation assumptions. Figure 1 Illustrates a symbiotic AI ecosystem.

**Figure 2:**
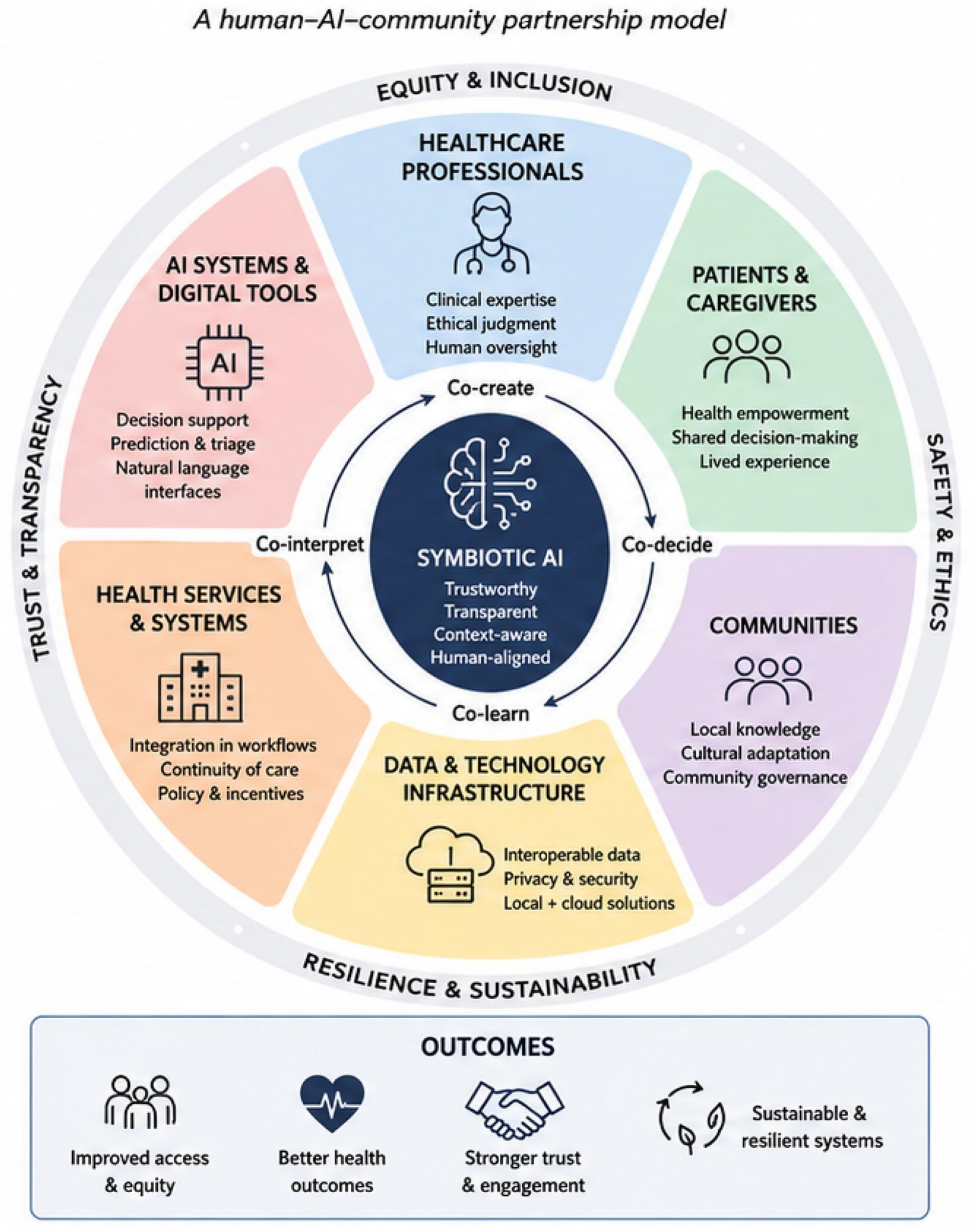
Symbiotic AI ecosystem for equitable digital health: A human–AI–community partnership model.

Figure 2 illustrates the core elements of a symbiotic AI ecosystem for equitable digital health. At the center of the model is symbiotic AI, characterized by trustworthiness, transparency, context-awareness, and human alignment. Surrounding this core are six interconnected domains: healthcare professionals, patients and caregivers, communities, data and technology infrastructure, health services and systems, and AI systems and digital tools. These domains are linked through continuous processes of co-creation, co-decision, co-interpretation, and co-learning. The outer layer emphasizes the guiding principles of equity and inclusion, trust and transparency, safety and ethics, and resilience and sustainability. The intended outcomes include improved access and equity, better health outcomes, stronger trust and engagement, and more sustainable and resilient health systems.

## Toward resilient symbiotic health ecosystems in rural and disrupted contexts

One of the least explored dimensions of digital health equity concerns healthcare resilience under conditions of infrastructural disruption. Rural and geographically isolated communities may experience periods in which avalanches, landslides, storms, flooding, or prolonged power outages significantly reduce access to healthcare services, transportation systems, electricity, mobile networks, and Internet connectivity.

Current digital health systems often depend heavily on centralized cloud infrastructures, stable broadband access, and uninterrupted electricity supply. Consequently, healthcare systems may become vulnerable precisely when healthcare needs intensify during crises or infrastructure failures. This raises important questions regarding how equitable healthcare delivery can be maintained under conditions of partial infrastructural collapse.

In this context, resilient *symbiotic health ecosystems* may represent a promising conceptual direction for future digital health development. Such ecosystems integrate healthcare professionals, local communities, digital infrastructures, AI-supported systems, and low-threshold healthcare technologies into adaptive and context-sensitive healthcare networks.

Potential components of such ecosystems may include telemedicine and video-supported emergency consultations, wearable monitoring technologies, low-risk diagnostic devices such as portable CRP measurement systems, electrocardiogram (ECG) machines, glucose meters, urine dipsticks, blood pressure monitors, and AI-assisted first-line situational assessments that support healthcare professionals during resource-constrained situations. Importantly, these technologies should not be understood as replacements for clinical expertise, but rather as supportive mechanisms that strengthen continuity of care and situational awareness.

The convergence of wearable monitoring, laboratory biomarkers, digital biomarkers, and AI-supported interpretation is supported by a rapidly expanding evidence base. Recent research demonstrates that wearable-derived physiological data can be integrated with biomarker and clinical information to support longitudinal monitoring, early disease detection, and more personalized healthcare approaches (16–18). However, much of the existing literature remains focused on proof-of-concept studies, monitoring accuracy, digital biomarker development, and early disease prediction rather than fully integrated healthcare ecosystems operating at scale. Consequently, wearable–laboratory—AI ecosystems should currently be viewed as an emerging implementation pathway with considerable potential, rather than as an established healthcare model requiring no further validation.

Similarly, local infrastructural preparedness may become increasingly important within future digital health ecosystems. Backup energy systems, including battery generators or diesel generators, may contribute to healthcare continuity during prolonged power outages. Local Wi-Fi infrastructures may support communication resilience when mobile networks fail. In situations involving Internet disruptions, local or edge-based AI systems may provide limited forms of healthcare guidance and decision support without relying entirely on cloud connectivity. Local or edge-based AI systems represent a promising but still emerging area of digital health innovation. Recent studies suggest that offline and edge-enabled AI solutions may support healthcare delivery in low-connectivity environments through local decision support, remote monitoring, and preliminary clinical assessment (19–21). However, most existing evidence is derived from proof-of-concept studies, pilot implementations, or technical evaluations rather than large-scale clinical deployment. Consequently, the clinical effectiveness, scalability, and long-term sustainability of edge-based AI systems remain insufficiently established and require further empirical evaluation.

**Figure 3:**
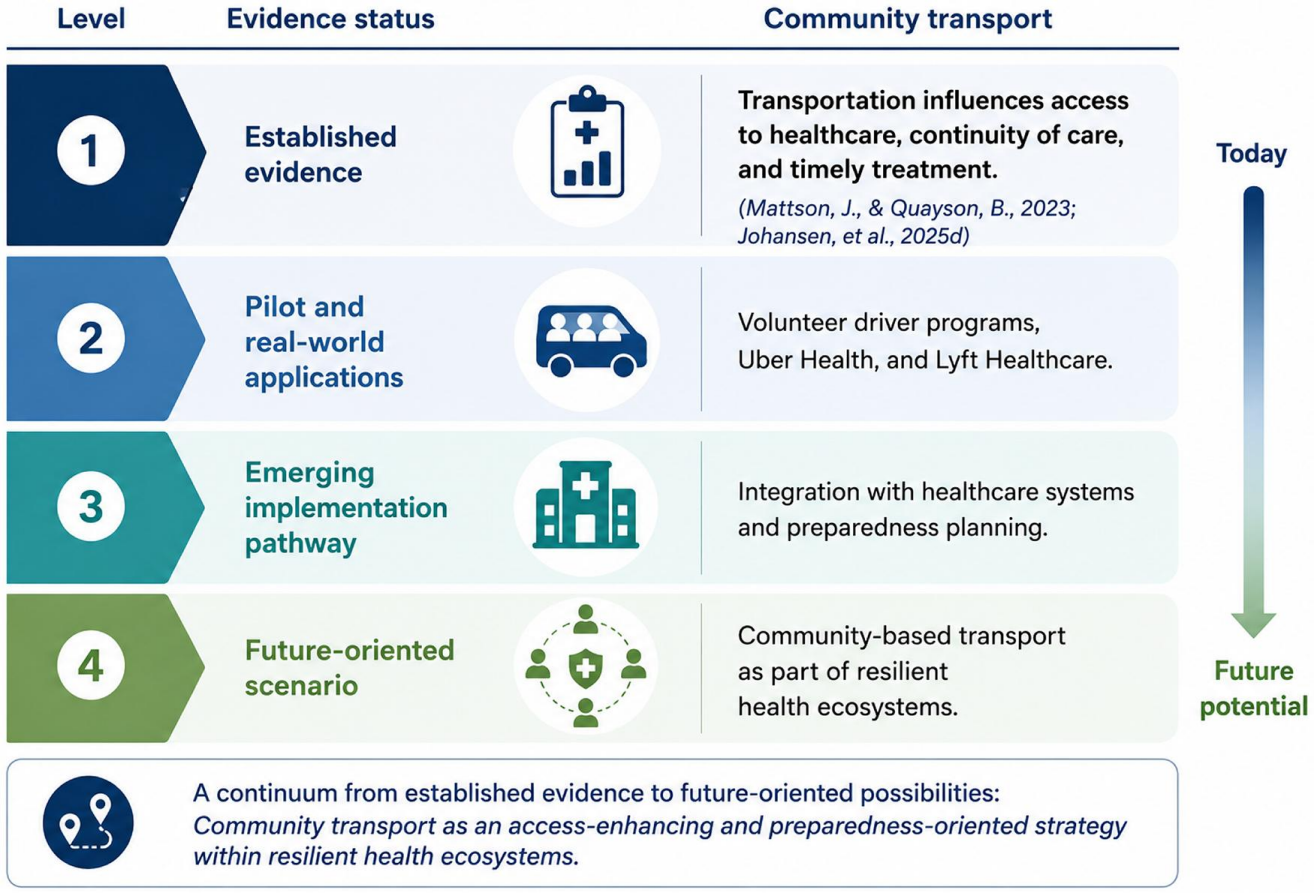
Community-supported transport systems.

These perspectives are particularly relevant in geographically isolated regions where weather conditions, natural hazards, or infrastructure fragility may disrupt transportation and communication systems. In such settings, community-supported healthcare models may also play a significant role. Local transport support systems inspired by community-based or sharing-economy principles may contribute to patient transportation during emergencies or infrastructure failures while complementing formal healthcare systems and emergency preparedness structures. However, community-supported transport systems should be viewed as a future-oriented preparedness and access strategy rather than as an established healthcare intervention (see Figure 3). Existing evidence demonstrates that transportation is a critical social determinant of health and that inadequate transport contributes to delayed care, missed appointments, and healthcare inequities, particularly in rural and underserved regions (22). Volunteer driver programs, community transportation services, and ridesharing models have increasingly been used to facilitate access to healthcare, while platforms such as Uber Health illustrate how transportation network services may support healthcare access in practice. However, robust evidence regarding their effectiveness as integrated healthcare interventions remains limited. Consequently, community-supported transport systems are best understood as promising access-enhancing and preparedness-oriented approaches that warrant further evaluation and implementation research. And such models might be developed with clear governance mechanisms, reimbursement structures, and coordination with public authorities, analogous to how voluntary emergency organizations may be compensated for public service contributions.

Drones may also become relevant in future rural and disrupted health ecosystems, particularly for the delivery of medicines, laboratory samples, or essential medical equipment when roads are blocked. Drone-based medicine delivery should not be viewed solely as a speculative future scenario. Jairoun et al. (23) demonstrate that medical drones are already being used for the delivery of medications, laboratory samples, blood products, and emergency supplies, particularly in rural and hard-to-reach areas. At the same time, the authors emphasize that important regulatory, logistical, and operational challenges remain to be addressed before wider implementation can be achieved. Similarly, Habibi et al. (24), in a systematic review of 136 studies (preprint), concluded that drones have the potential to reduce response times, improve access to critical medical supplies, and provide particular benefits in disaster-prone and rural settings. However, they also highlighted the need for further research on safety, regulatory frameworks, and large-scale implementation. Despite these promising developments, the field still lacks large-scale studies evaluating the effectiveness, scalability, and sustainability of drone-based delivery systems for transporting medicines, blood products, diagnostic samples, and emergency equipment in remote and disaster-prone regions. Furthermore, wider implementation remains contingent upon regulatory approval, logistical capacity, and supporting infrastructure.

In the future such solutions must be embedded in regulated, clinically supervised, and locally coordinated preparedness systems. Similarly, voluntary access to laboratory data, such as data from established laboratory providers, may support more informed clinical assessments when combined with local measurements, wearable data, and video-supported consultations.

AI-assisted image analysis may further support first-line situational assessment in disrupted contexts. For example, images of injuries, rashes, swelling, trauma, or other visible symptoms may be analyzed by AI systems as a preliminary support mechanism before healthcare professionals conduct more thorough clinical assessments through video consultation or other communication channels. AI-supported image triage should be considered an emerging implementation area rather than a purely hypothetical future scenario. In the United Kingdom, the National Institute for Health and Care Excellence (25) has granted conditional approval for the use of artificial intelligence–based skin cancer triage technologies, while further evidence regarding their clinical effectiveness, safety, and implementation is being gathered. Pilot studies in dermatology and wound care have demonstrated promising diagnostic and triage performance, although further evidence regarding effectiveness, equity, and large-scale implementation remains necessary (26).

Such use of AI-supported image triage must be framed as supportive triage and situational awareness rather than autonomous diagnosis. Human oversight, clear accountability, and clinical escalation pathways remain essential.

Krumsvik et al. (27) suggested that AI-supported healthcare ecosystems may strengthen health empowerment and preparedness in avalanche- or landslide-isolated rural communities. Similarly, Johansen et al. (28) demonstrated how earlier communication and intervention may significantly improve time-sensitive healthcare outcomes such as cardiac arrest survival. Together, these developments point toward a broader conceptual shift from isolated digital health technologies toward integrated and resilient symbiotic healthcare ecosystems.

Importantly, resilience should not be reduced to technological robustness alone. Resilient healthcare ecosystems also depend on social trust, local participation, healthcare literacy, institutional coordination, and adaptive governance structures. Consequently, equitable digital health requires not only technological innovation, but also community-centered implementation strategies capable of functioning under varying infrastructural and societal conditions.

**Figure 4:**
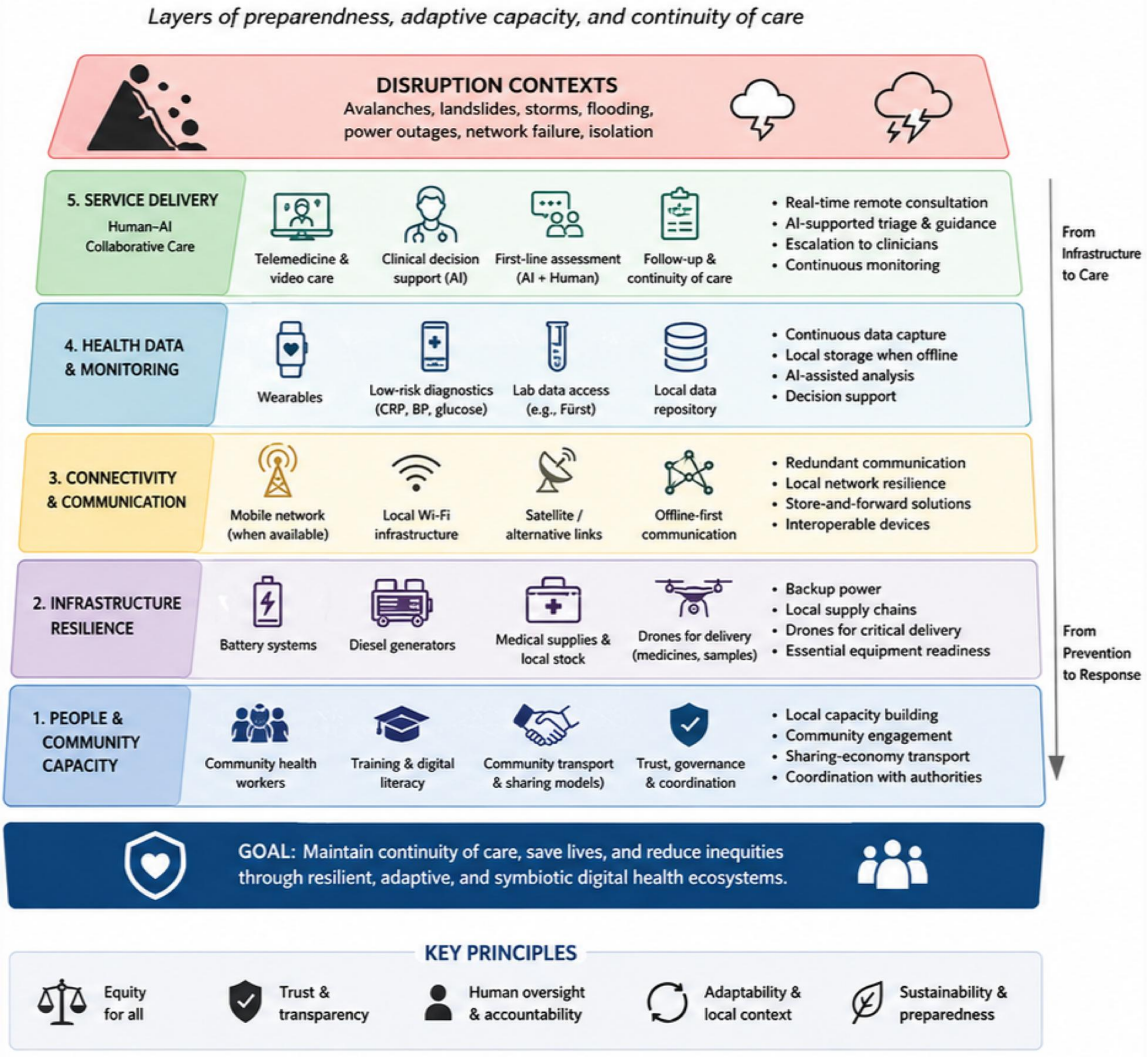
Resilient digital health ecosystem for rural and disrupted contexts: layers of preparedness, adaptive capacity, and continuity of care.

Figure 4 presents a layered model of a resilient digital health ecosystem designed for rural and disrupted contexts. The model begins with disruption contexts such as avalanches, landslides, storms, flooding, power outages, network failures, and geographical isolation. It then outlines five interdependent layers of preparedness and response. The foundational layer concerns people and community capacity, including community health workers, training and digital literacy, community transport and sharing models, trust, governance, and coordination. The second layer concerns infrastructure resilience, including battery systems, diesel generators, local medical supplies, local stockpiles, and drones for critical delivery of medicines, samples, or equipment. The third layer concerns connectivity and communication, including mobile networks when available, local Wi-Fi infrastructure, satellite or alternative links, offline-first communication, and store-and-forward solutions. The fourth layer concerns health data and monitoring, including wearables, low-risk diagnostics, voluntary access to laboratory data, local data repositories, continuous data capture, and AI-assisted analysis. The fifth layer concerns service delivery, including telemedicine and video care, clinical decision support, AI-supported first-line assessment, follow-up, continuity of care, and escalation to clinicians when necessary. Across these layers, the ecosystem is guided by principles of equity for all, trust and transparency, human oversight and accountability, adaptability to local context, and sustainability and preparedness. The overarching goal is to maintain continuity of care, save lives, and reduce inequities through resilient, adaptive, and symbiotic digital health ecosystems.

## Wearable technologies, longitudinal physiological monitoring, laboratory biomarkers, and AI-supported decision-support systems

Over the past decade, wearable technologies such as smartwatches, fitness trackers, and smart rings have become increasingly integrated into everyday life. Recent evidence suggests that wearable ownership is now widespread, with approximately 44%–53% of adults in large population surveys reporting ownership of at least one wearable health-tracking device (29–32). Globally, hundreds of millions of individuals routinely generate continuous streams of physiological and behavioural data through wearable devices, creating unprecedented opportunities for longitudinal health monitoring beyond traditional healthcare encounters.

From the perspective of symbiotic intelligence, the significance of wearable technologies extends beyond the devices themselves. Wearables introduce a new layer of continuously generated real-world data that complements the episodic point-in-time assessments traditionally obtained through clinical consultations and laboratory testing. While laboratory biomarkers and professional clinical assessments remain the most reliable and clinically validated sources of health information, wearable technologies provide longitudinal observations of everyday physiological processes occurring between healthcare encounters. Consequently, wearable-derived data should not be viewed as a replacement for professional assessment but as a complementary source of information that may contribute to a more dynamic, person-centred, and context-sensitive understanding of health.

Advanced wearables are now capable of continuously monitoring physiological parameters such as resting heart rate, heart rate variability, sleep patterns, oxygen saturation, stress responses, recovery, and physical activity (33). Emerging evidence suggests that certain wearable-derived indicators may be particularly informative in this regard. For example, Esmaeilpour et al. (34) found that step count, Active Zone Minutes, and resting heart rate emerged as some of the strongest wearable-derived predictors associated with cardiovascular and metabolic deterioration. Similar associations have been reported in other studies examining physical activity, resting heart rate, cardiorespiratory fitness, and cardiometabolic risk profiles (35).

When such longitudinal data streams are combined with laboratory biomarkers, including inflammatory markers, metabolic indicators, blood pressure, and cardiovascular risk profiles, AI-supported systems may contribute to earlier situational awareness, more individualized preventive strategies, and strengthened health empowerment (36). Importantly, the value of such systems does not primarily emerge from isolated measurements, but from the interpretation of multimodal physiological patterns over time.

Recent research within digital biomarkers and wearable technologies increasingly demonstrates how longitudinal wearable-data can be integrated with physiological and clinical biomarkers to support health monitoring, prevention, and early disease detection (33). Studies on heart rate variability (HRV) further suggest that autonomic regulation is closely associated with inflammatory processes and physiological resilience (37), which is particularly relevant given that modern wearables can now monitor HRV continuously over time. Similarly, extensive evidence from cardiovascular research demonstrates that high cardiorespiratory fitness (CRF) is strongly associated with lower cardiovascular risk, improved metabolic health, and reduced all-cause mortality (38). For example, the large-scale JAMA cohort study by Mandsager et al. showed that higher fitness levels were associated with progressively lower mortality risk without any clearly observed upper threshold for benefit (39). Meta-analyses also indicate that elevated resting heart rate is associated with increased risk of cardiovascular disease and overall mortality (40), suggesting that longitudinal monitoring of resting heart rate and autonomic regulation may function as clinically relevant biomarkers over time. Mun et al. (41) find similar tendencies and that resting heart rate can be monitored by wearables for early identification of metabolic syndrome risk.

However, although the growing knowledge base suggests considerable unrealized potential for consumer wearables in cardiovascular medicine, the field is also characterized by several important challenges and limitations related to validity, interpretability, implementation, data quality, GDPR, and clinical integration (42, 43).

Together, these developments point toward a broader shift in which the boundaries between clinical medicine, everyday life, and digital health technologies may gradually become less distinct—especially in rural communities with limited access to medical expertise and primary health care. Large-scale studies increasingly demonstrate robust associations between wearable-derived measures such as daily step counts, resting heart rate, and combined wearable-based indicators of physical activity, activity intensity, and physiological recovery, and the risk of cardiovascular and metabolic dysfunction. As wearable technologies, real-world data, laboratory biomarkers, and AI-supported systems increasingly begin to “communicate” across integrated health ecosystems, future healthcare systems may gain access to substantially more dynamic, individualized, and longitudinal understandings of human health than previously possible.

This development may also contribute to a broader conceptual shift from chronologically oriented healthcare models toward more functional and biologically informed understandings of health, ageing, resilience, and recovery capacity. In this context, AI-supported systems may increasingly function as supportive analytical layers capable of identifying subtle physiological changes associated with stress, inflammation, metabolic dysregulation, or early disease development before clinical symptoms become apparent.

At the same time, these developments raise important questions related to privacy, data ownership, algorithmic transparency, overdiagnosis, commercialization of health data, and unequal access to advanced health technologies. Consequently, future implementation efforts must ensure that wearable ecosystems and AI-supported health infrastructures strengthen human autonomy, contextual interpretation, and equitable healthcare delivery rather than contributing to increased surveillance, technological dependency, or reductionist understandings of human health (44).

Within ageing societies characterized by increasing healthcare demands, workforce shortages, and growing pressure on primary healthcare systems, preventive and health-empowering approaches may become increasingly important. Symbiotic AI ecosystems integrating wearable technologies, laboratory data, telemedicine, and human clinical oversight may therefore contribute not only to more personalized healthcare, but also to more sustainable and resilient healthcare systems capable of supporting continuity of care across diverse populations and infrastructural conditions. But how might such scenarios unfold in real life in the coming years?

**Figure 5:**
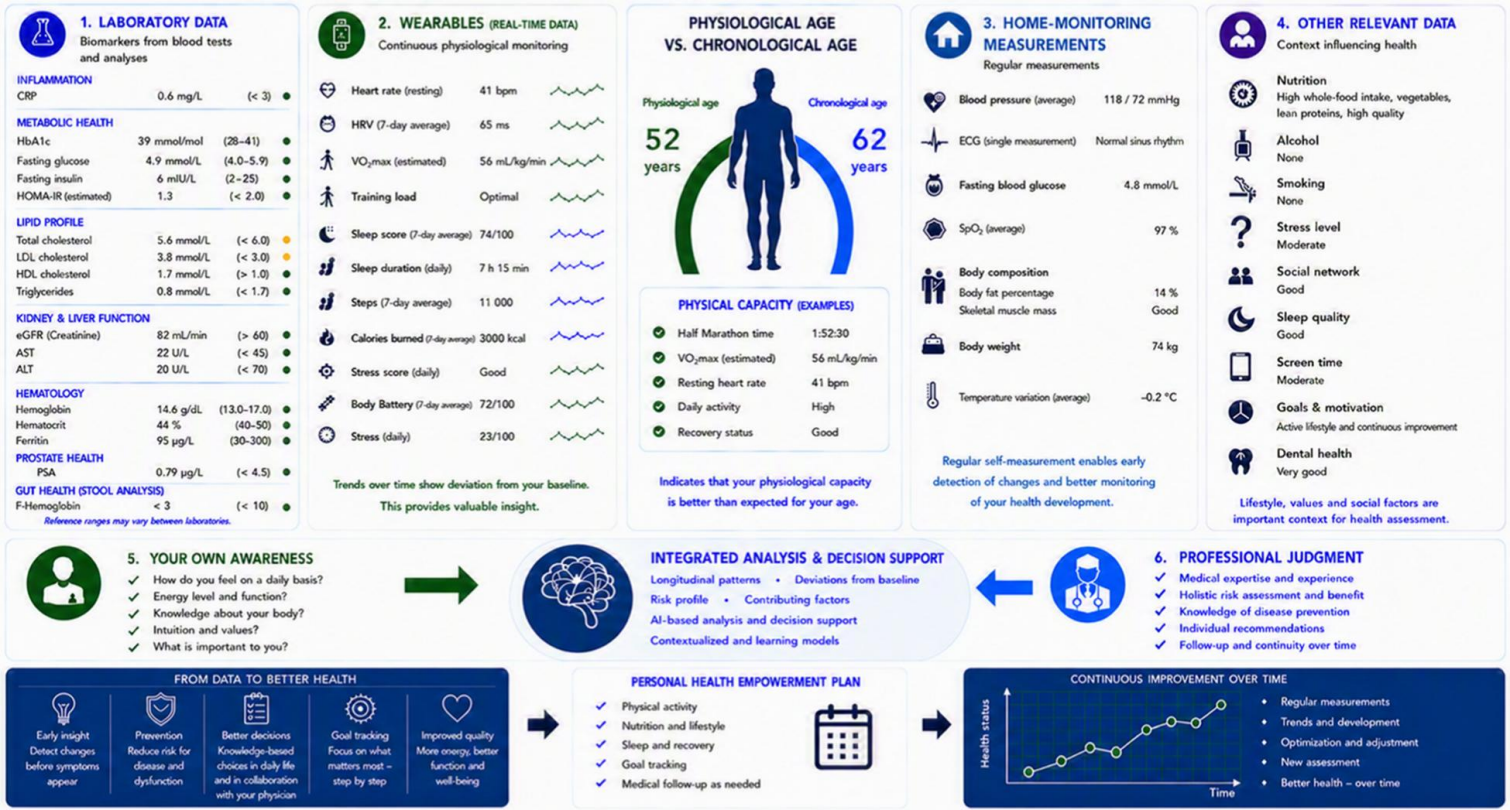
Symbiotic intelligence ecosystem and decision support.

Figure 5 illustrates this through a fictitious example of a symbiotic intelligence ecosystem at a concrete level, highlighting the interplay between different biomarkers and health data for a 62-year-old man living in a rural community without access to nurses, doctors, dentists, or other healthcare expertise in the local village. In a symbiotic intelligence ecosystem, the integration of laboratory data, wearable data, home-based measurements, lifestyle factors, personal judgment, and physicians' professional assessments can contribute to a more holistic and dynamic understanding of health over time. Within such an ecosystem, one might to some extent reinterpret conventional understandings of human age and health. The figure illustrates how different sources of health information contribute complementary forms of knowledge within a symbiotic intelligence ecosystem. Importantly, the various components differ in terms of reliability, risk, temporal resolution, and dependence on human judgment.

Laboratory biomarkers (1) and professional clinical assessments (6) may be regarded as the primary gatekeepers within the ecosystem. Their strength lies in relatively high reliability, established clinical validity, and low risk of misinterpretation when conducted and interpreted according to professional standards. However, both are typically based on episodic or point-in-time measurements and therefore provide only snapshots of an individual's health status.

Wearable monitoring and real-world physiological data (2) provide a different type of insight. Their primary strength is the continuous collection of longitudinal biomarker and behavioural data under real-life conditions, often on a 24-hour basis over extended periods. Such data may capture physiological changes, recovery patterns, physical activity, sleep, and autonomic regulation that are difficult to identify through isolated clinical measurements alone. However, wearable-derived data generally have lower clinical reliability and validity than laboratory analyses and professional assessments and should therefore be interpreted cautiously and preferably in conjunction with other sources of information.

Home-based measurements and local health hubs (3) occupy an intermediate position within the ecosystem. When reliable equipment is available, such measurements may provide clinically relevant information with relatively low risk and reasonable reliability. However, they remain dependent on access to appropriate devices, local infrastructure, or community-based health resources, and are typically based on intermittent rather than continuous measurements.

Self-reported symptoms and health experiences (4) represent an important source of contextual information but are inherently more vulnerable to recall bias, misunderstanding, subjective interpretation, and reporting inaccuracies. Consequently, their reliability may vary substantially across individuals and situations.

Similarly, self-reflection, lifestyle reporting, and personal health narratives (5) play a central role in health empowerment, self-awareness, and patient engagement. These dimensions are essential for understanding behaviours, motivations, and lived experiences that may not be captured through objective measurements alone. At the same time, they are particularly susceptible to cognitive biases, social desirability effects, incomplete reporting, and varying levels of health literacy.

The central premise of the symbiotic intelligence ecosystem is therefore not that any single source provides a complete representation of health. Rather, value emerges through the integration and triangulation of complementary data sources, where objective clinical measurements, continuous physiological monitoring, contextual self-reporting, and professional judgment collectively contribute to a more holistic, dynamic, and person-centred understanding of health over time.

Clinical consultations and laboratory testing remain the most reliable and clinically validated sources of health information. However, they typically represent relatively infrequent observations of an individual's health status. In Norway, individuals aged 50–66 years have on average approximately 3.5 consultations with their general practitioner annually, and 77% have at least one consultation during the year. Furthermore, men generally utilize primary healthcare services less frequently than women, with the Norwegian Institute of Public Health reporting approximately 1.2–1.25 fewer annual consultations among men. In addition, around 16% (175,000–185,000) of Norwegian men aged 20–50 years had not consulted either a general practitioner or emergency primary care service during a three-year period (45–47). Several explanations have been proposed for men's comparatively lower utilization of primary healthcare services, especially those residing in rural and non-urban areas (10). A systematic review finds that these include traditional masculine norms emphasizing self-reliance, stoicism, and reluctance to seek help, as well as gender-related stereotypes regarding illness, vulnerability, and health-seeking behaviour (48). A scoping review finds that the healthcare system may not always engage men effectively and that some men may delay healthcare utilization until symptoms become more severe, while organizational and relational factors within healthcare systems may further influence engagement (49). In some cases, paternalistic communication styles or perceived lack of patient involvement in clinical encounters may reduce motivation for preventive consultations and follow-up. Persson et al. (50) finds that gendered expectations within healthcare encounters may influence men's healthcare engagement. Consequently, differences in healthcare utilization are likely shaped by a complex interaction of individual, cultural, social, and healthcare-system factors rather than by any single explanation alone.

Although clinical consultations and laboratory testing remain the most reliable sources of health information, they typically represent only a limited number of observations each year. Wearable technologies, in contrast, may provide continuous physiological monitoring between healthcare encounters, thereby complementing rather than replacing professional assessment.

These findings suggest that even highly reliable clinical assessments and laboratory measurements often provide only episodic snapshots of an individual's health. By contrast, wearable technologies may generate continuous streams of physiological and behavioural data throughout daily life, potentially capturing health-related changes that occur between healthcare encounters. Consequently, the distinction between clinical assessments and wearable monitoring should not primarily be understood as a competition between superior and inferior data sources, but rather as a trade-off between high validity and low temporal resolution on the one hand, and lower validity but continuous longitudinal observation on the other. Within a symbiotic intelligence ecosystem, the value emerges from integrating these complementary forms of knowledge rather than relying on any single source in isolation.

## Risks and unintended consequences

While symbiotic AI ecosystems may offer substantial opportunities for improving healthcare accessibility, resilience, and health empowerment, they also introduce important risks and unintended consequences that require careful consideration. Algorithmic bias may contribute to unequal healthcare outcomes if AI systems are trained on unrepresentative datasets or insufficiently validated across diverse populations. Increased use of wearable technologies, continuous monitoring, and AI-supported decision systems may also raise concerns related to surveillance, privacy, and individual autonomy. Furthermore, growing commercialization of digital health platforms may create tensions between public health objectives and commercial interests, particularly regarding the ownership, governance, and secondary use of personal health data.

Additional concerns include the potential for overdiagnosis and unnecessary healthcare utilization resulting from continuous monitoring and highly sensitive detection systems. Questions of liability and accountability also remain unresolved when clinical decisions are influenced by AI-supported recommendations. Finally, regulatory uncertainty continues to challenge the implementation of emerging digital health technologies, as governance frameworks often struggle to keep pace with rapid technological developments. Consequently, the future development of symbiotic AI ecosystems must be accompanied by robust governance structures, transparent data practices, human oversight, and equity-centered regulatory frameworks capable of balancing innovation with patient safety, trust, and societal accountability.

## Operationalization and future research

Although the five figures presented in this Perspective are conceptual in nature, they are intended to generate empirically testable propositions for future research. The proposed frameworks may be examined through mixed-methods research designs combining quantitative indicators with qualitative investigations of implementation processes, contextual adaptation, and user experiences.

For example, Figure 1 highlights the need for further conceptual clarification and theoretical development within this emerging field. Figure 2 (Symbiotic AI Ecosystem) may be evaluated through measures of trust in AI-supported healthcare, patient satisfaction, healthcare accessibility, clinician acceptance, and health outcomes. Figure 3 (Community-Supported Transport Systems) may be assessed through indicators such as healthcare access, missed appointments, continuity of care, and response times in underserved communities. Figure 4 (Resilient Digital Health Ecosystem) may be examined through measures of service availability, continuity of care during infrastructure disruptions, preparedness capacity, and system resilience. Figure 5 (Symbiotic Intelligence Ecosystem for Health Empowerment and Decision Support) may be investigated through indicators of health literacy, patient engagement, self-management, adherence, and preventive health behaviours.

Methodologically, future research may benefit from implementation science approaches, longitudinal cohort studies, case studies, and mixed-methods designs capable of capturing both measurable outcomes and contextual mechanisms. Such approaches may help evaluate how symbiotic AI ecosystems influence equity, trust, resilience, and health empowerment across diverse healthcare settings.

## Discussion

This *Perspective* has argued that equitable digital health requires more than technological access alone. Emerging real-world usage patterns suggest that generative AI is increasingly functioning as a complementary healthcare navigation layer, particularly in underserved and geographically isolated settings (9, 13). Future healthcare systems must also address trust, resilience, contextual adaptation, implementation feasibility, infrastructural vulnerability, and health literacy. Within this landscape, symbiotic AI ecosystems may provide a promising conceptual framework for understanding how human expertise and AI systems can collaboratively support equitable healthcare delivery.

Importantly, the proposed perspective does not advocate technological determinism or replacement of healthcare professionals. Rather, it emphasizes trustworthy augmentation, calibrated trust, human oversight, and context-sensitive implementation strategies. In this sense, the future of equitable digital health may depend less on autonomous AI systems and more on how AI becomes integrated into broader human-centered healthcare ecosystems.

The article further highlights the importance of resilience-oriented healthcare infrastructures in underserved and disruption-prone settings. As climate-related events, infrastructure vulnerabilities, and geopolitical instability increasingly affect societies worldwide, digital health systems should increasingly be designed not only for efficiency, but also for preparedness, continuity, and adaptability under adverse conditions.

Several implications emerge from this discussion. First, future digital health policies should increasingly incorporate equity-centered and resilience-oriented implementation frameworks. Second, research on AI in healthcare should move beyond narrow performance metrics and include broader questions related to trust, epistemic transparency, contextual adaptation, and long-term sustainability. Third, healthcare systems may benefit from exploring hybrid and community-supported healthcare models integrating telemedicine, local infrastructures, low-threshold diagnostics, wearable technologies, AI-supported guidance, backup energy systems, local communication networks, and coordinated transport support within broader preparedness strategies (as shown in Figures 1–5). Fourth, given the complexity of symbiotic AI ecosystems, mixed-methods approaches may be particularly valuable for understanding both measurable outcomes and the contextual mechanisms through which trust, resilience, and health empowerment emerge.

Beyond technological innovation, the successful implementation of symbiotic AI ecosystems will depend upon broader questions of governance, institutional adaptation, and societal capacity to manage digital transformation. As suggested by emerging governance perspectives, the long-term sustainability of digital health systems requires not only technological advancement but also adaptive regulatory frameworks, public trust, ethical oversight, and mechanisms for balancing innovation with societal needs (51–53). From this perspective, resilient health ecosystems may be understood not merely as technological infrastructures but as socio-technical systems shaped by governance, culture, institutions, and community participation.

Finally, the concept of *symbiotic intelligence* may contribute to reframing ongoing debates about AI in healthcare. Rather than understanding AI as either a threat or a solution in isolation, future research and implementation efforts should increasingly explore how trustworthy and context-aware human—AI collaboration can strengthen equitable healthcare ecosystems across diverse populations and settings. Ultimately, digital health equity is not achieved through technology alone, but through trusted, resilient, inclusive, and locally adaptive health ecosystems that place humans and communities at the centre.

## Data Availability

The original contributions presented in the study are included in the article/Supplementary Material, further inquiries can be directed to the corresponding author.
